# Supplementary material for: exRNA-eCLIP intersection analysis reveals a map of extracellular RNA binding proteins and associated RNAs across major human biofluids and carriers
Source: Cell Genom. 2023 Apr 20;3(5):100303. doi: 10.1016/j.xgen.2023.100303 (PMC10203258; doi:10.1016/j.xgen.2023.100303)
Supplement: Document S1. Figures S1–S4 and Tables S1–S4 [file mmc1.pdf]

Cell Genomics, Volume *3*

Supplemental information

exRNA-eCLIP intersection analysis reveals a map of extracellular RNA binding proteins and associated RNAs across major human biofluids and carriers

Emily L. LaPlante, Alessandra Stürchler, Robert Fullem, David Chen, Anne C. Starner, Emmanuel Esquivel, Eric Alsop, Andrew R. Jackson, Ionita Ghiran, Getulio Pereira, Joel Rozowsky, Justin Chang, Mark B. Gerstein, Roger P. Alexander, Matthew E. Roth, Jeffrey L. Franklin, Robert J. Coffey, Robert L. Raffai, Isabelle M. Mansuy, Stavros Stavrakis, Andrew J. deMello, Louise C. Laurent, Yi-Ting Wang, Chia-Feng Tsai, Tao Liu, Jennifer Jones, Kendall Van Keuren-Jensen, Eric Van Nostrand, Bogdan Mateescu, and Aleksandar Milosavljevic

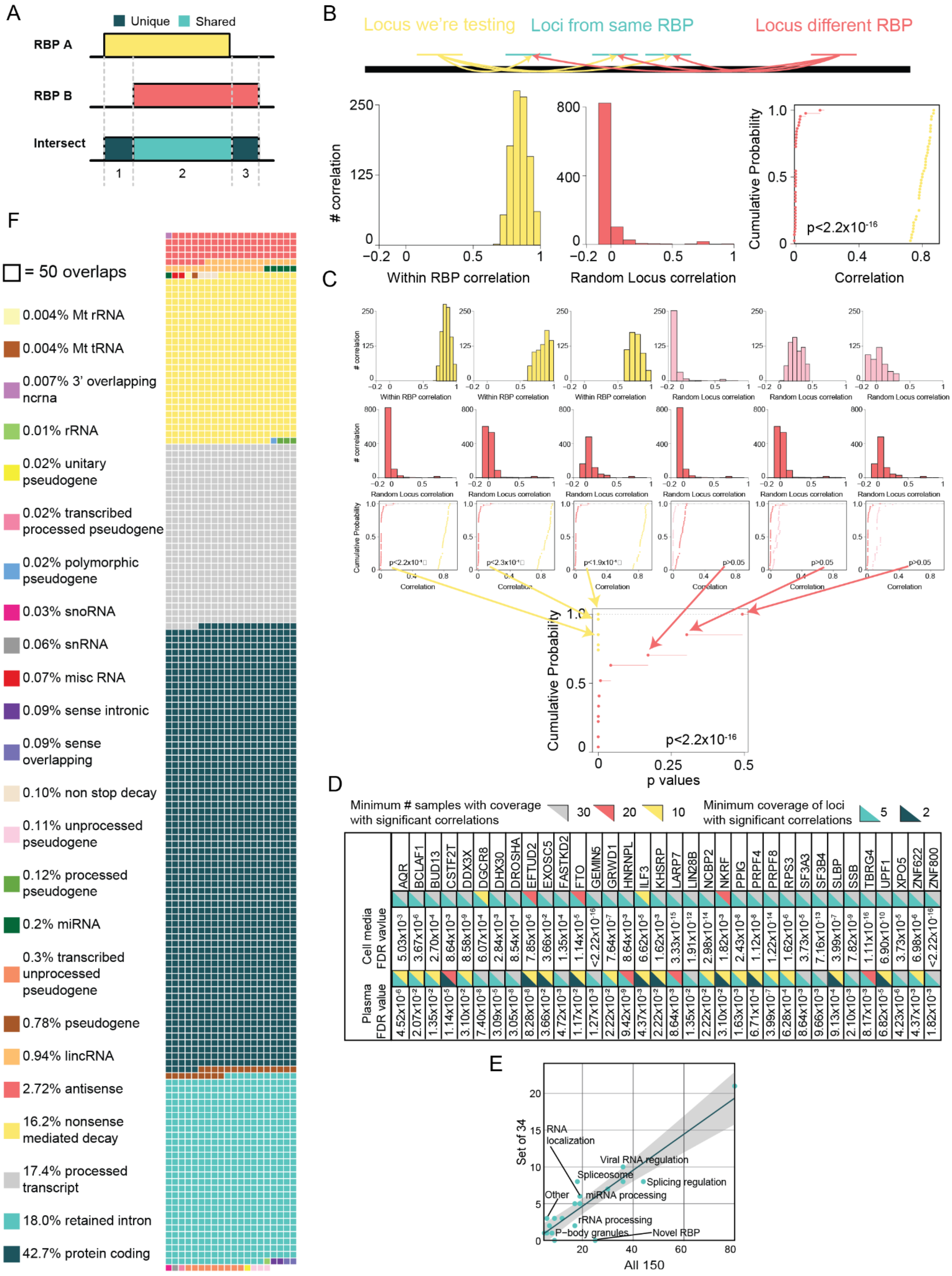

	Cell media FDR vavlue	Plasma FDR value
AQR	$5.03x10^{-3}$	$4.52x10^{-6}$
BCLAF1	$3.67x10^{-6}$	$2.07x10^{-2}$
BUD13	$2.70x10^{-4}$	$1.35x10^{-2}$
CSTF2T	$8.64x10^{-3}$	$1.14x10^{-5}$
DDX3X	$8.58x10^{-9}$	$3.10x10^{-2}$
DGCR8	$6.07x10^{-4}$	$7.40x10^{-8}$
DHX30	$2.84x10^{-3}$	$3.09x10^{-5}$
DROSHA	$8.54x10^{-4}$	$3.05x10^{-8}$
EFTUD2	$7.85x10^{-6}$	$8.28x10^{-8}$
EXOSC5	$3.66x10^{-2}$	$3.66x10^{-2}$
FASTKD2	$1.35x10^{-4}$	$4.72x10^{-3}$
FTO	$1.14x10^{-5}$	$1.17x10^{-2}$
GEMIN5	$<2.22x10^{-16}$	$1.27x10^{-3}$
GRWD1	$7.64x10^{-7}$	$2.22x10^{-2}$
HNRNPL	$8.64x10^{-3}$	$9.42x10^{-9}$
ILF3	$6.62x10^{-5}$	$4.37x10^{-3}$
KHSRP	$1.62x10^{-3}$	$2.22x10^{-2}$
LARP7	$3.33x10^{-15}$	$8.64x10^{-3}$
LIN28B	$1.91x10^{-12}$	$1.35x10^{-2}$
NCBP2	$2.98x10^{-14}$	$2.22x10^{-2}$
NKRF	$1.82x10^{-3}$	$3.10x10^{-2}$
PPIG	$2.43x10^{-8}$	$1.63x10^{-3}$
PRPF4	$1.12x10^{-8}$	$6.71x10^{-4}$
PRPF8	$1.22x10^{-14}$	$3.99x10^{-7}$
RPS3	$1.62x10^{-6}$	$6.28x10^{-4}$
SF3A3	$3.73x10^{-5}$	$8.64x10^{-3}$
SF3B4	$7.16x10^{-13}$	$9.66x10^{-3}$
SLBP	$3.99x10^{-7}$	$9.13x10^{-4}$
SSB	$7.82x10^{-9}$	$2.10x10^{-3}$
TBRG4	$1.11x10^{-16}$	$8.17x10^{-3}$
UPF1	$6.90x10^{-10}$	$6.82x10^{-5}$
XPO5	$3.73x10^{-5}$	$4.23x10^{-6}$
ZNF622	$6.98x10^{-6}$	$4.37x10^{-3}$
ZNF800	$<2.22x10^{-16}$	$1.82x10^{-3}$

Figure S1

Kolmogorov–Smirnov tests identify RBPs with significantly correlated loci, Related to Figure 1. A) An example of RBP intersections – intersecting two RBPs leads to 4 regions, 3 unique and 1 shared. **B)** Taking the distribution of a single locus and correlating it to all other loci from the same RBP. That distribution is compared to the correlations from a random locus with similar coverage to the same set of loci from the original RBP. Kolmogorov–Smirnov tests determine if the locus is significantly correlated. **C)** P values from all loci of a given RBP created using the method from Figure S1B are compared to p values generated by comparing correlation distributions of all the random loci (left 3 plots). The distribution of the random locus correlated to the RBP loci is also compared against the distribution of a random locus correlated to random loci to create the second distribution of p values (right 3 plots). Kolmogorov–Smirnov tests determine if the p value of all loci in an RBP distribution are significantly different than random to determine an RBP level metric. **D)** Kolmogorov–Smirnov test Bonferroni adjusted p values for RBPs significant in both cell media and human plasma. **E)** Linear regression between the number of RBPs with Kolmogorov–Smirnov test Bonferroni adjusted p values < 0.05 in both cell media and human plasma and the total number of RPBs in that category. Labeled categories fall outside the regression line. **F)** Gencode annotations that intersect with regions bound by the 34 RBPs. 1 box = 50 overlaps.

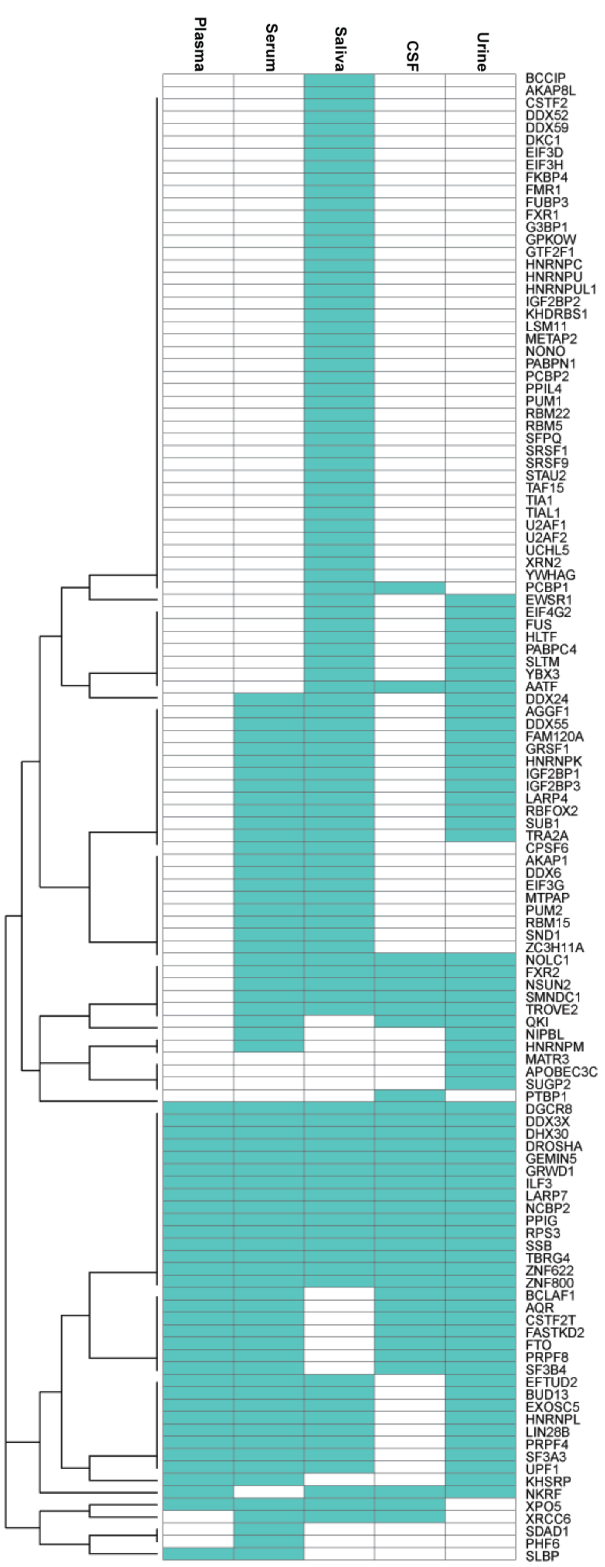

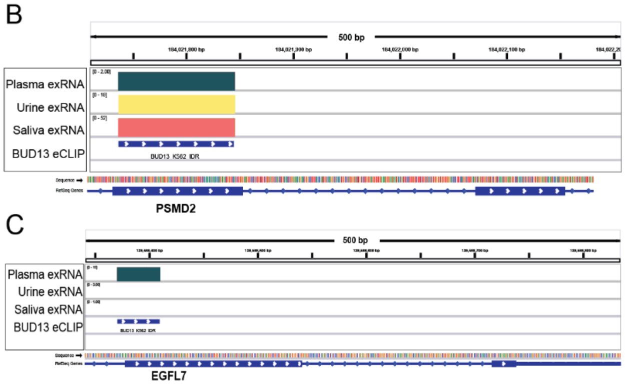

D

RBP	Correlation Footprint Unique RBP Loci	Correlation Footprint All RBP Loci	Detected in Condition Media Western Blot?
AATF	Yes	No	Not tested
BUD13	No	No	No
DGCR8	Yes	No	Yes
DHX30	Yes	No	Not tested
DROSHA	Yes	No	Not tested
EFTUD2	No	No	Yes
EXOSC5	No	Yes	Not tested
FASTKD2	Yes	No	Not tested
FTO	Yes	No	Yes
FUS	No	Yes	Not tested
FXR2	Yes	No	Not tested
GEMIN5	Yes	No	Not tested
GRSF1	No	Yes	Not tested
HNRNPK	No	Yes	Not tested
HNRNPM	No	Yes	Not tested
IGF2BP1	No	No	Not tested
IGF2BP3	No	Yes	Not tested
ILF3	Yes	No	Yes
KHSRP	No	Yes	Not tested
LARP4	No	No	Not tested
LARP7	Yes	Yes	Not tested
LIN28B	Yes	No	Yes
MATR3	No	Yes	Not tested
NCBP2	Yes	No	Yes
NKRF	Yes	No	Not tested
NOLC1	Yes	No	Not tested
NSUN2	Yes	Yes	Not tested
PCBP1	Yes	No	Not tested
PPIG	Yes	Yes	No
PRPF4	No	No	Not tested
PRPF8	No	No	Yes
PTBP1	Yes	No	Not tested
PUM2	No	No	Not tested
QKI	Yes	No	Not tested
RBFOX2	No	No	Not tested
RPS3	Yes	No	Yes
SF3A3	No	No	Yes
SF3B4	Yes	No	Yes
SLTM	Yes	No	Not tested
SMNDC1	Yes	No	Not tested
SND1	No	No	Not tested
SSB	Yes	Yes	Yes
SUB1	No	No	Not tested
TBRG4	Yes	Yes	Not tested
TRA2A	No	No	Not tested
TROVE2	Yes	No	Not tested
U2AF2	No	No	Not tested
UPF1	No	Yes	Not tested
XPO5	Yes	No	Yes
XRCC6	Yes	No	Not tested
XRN2	Yes	No	Not tested
ZNF622	Yes	No	Yes
ZNF800	Yes	No	Not tested

Figure S2

Results of exRBP Correlation Footprinting Analysis, Related to Figure 2. A) The RBPs that were detected by correlation footprinting in each analyzed biofluid. **B)** Example Integrative Genomic Viewer trace of a BUD13 eCLIP binding locus used for correlation footprinting, here exRNA samples show expression across all biofluids. **C)** Example Integrative Genomic Viewer trace of a BUD13 eCLIP binding locus used for correlation footprinting, here exRNA samples show biofluid specific expression only in plasma. **D)** Comparison between correlation footprinting methods in CSF across 53 RBPs. Showing results for when either filtering to only loci unique to an RBP before footprinting (column 2) or including all loci of an RBP when footprinting (column 3). And how those results compare to the western blot validation results for conditioned media (column 4).

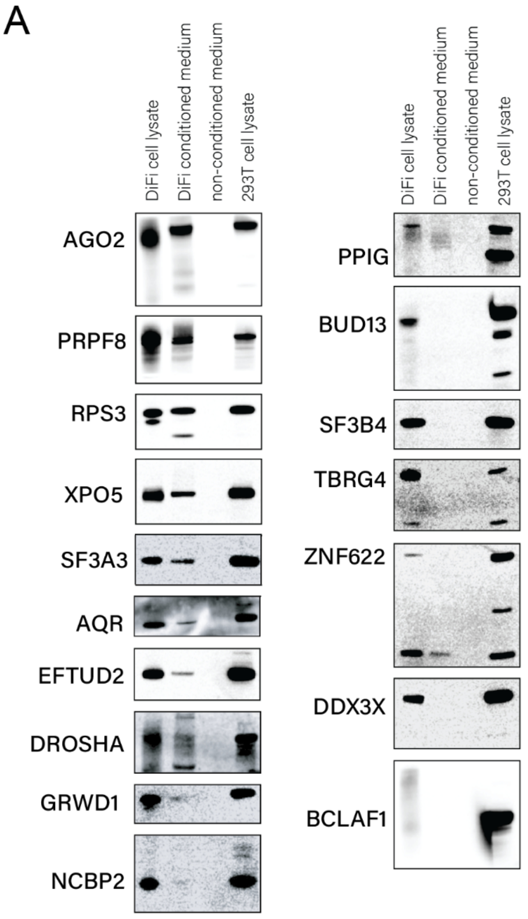

B

RBP	293T cond. media WB	hMSC cond. media WB	DiFi cond. media WB	Plasma mass spectrometry
AQR	Detected	Not expressed	Detected	Detected
BCLAF1	Not detected	Not expressed	No detected	Detected
BUD13			No detected	Not detected
CSTF2T				Not detected
DDX3X	Not detected	Not detected	No detected	Detected
DGCR8	Detected	Not detected		Detected
DHX30				Not detected
DROSHA				Detected
EFTUD2	Detected	Detected	Detected	Detected
EXOSC5				Detected
FASTKD2				Not detected
FTO	Detected	Detected		Detected
GEMIN5				Detected
GRWD1	Detected	Detected	Detected	Detected
HNRNPL				Detected
ILF3	Detected	Detected		Detected
KHSRP				Detected
LARP7				Not detected
LIN28B	Detected	Not expressed		Not detected
NCBP2	Detected	Not detected	Detected	Detected
NKRF				Detected
PPIG	Not detected	Not expressed	Not expressed	Detected
PRPF4				Detected
PRPF8	Not detected	Detected	Detected	Detected
RPS3	Detected	Not detected	Detected	Detected
SF3A3	Detected	Detected	Detected	Detected
SF3B4	Detected	Detected	No detected	Detected
SLBP				Detected
SSB	Detected	Detected		Not detected
TBRG4			No detected	Not detected
UPF1				Detected
XPO5	Detected	Detected	Detected	Not detected
ZNF622	Detected	Not expressed	No detected	Detected
ZNF800				Not detected
	14/18	9/18	8/15	24/34
	27/34			

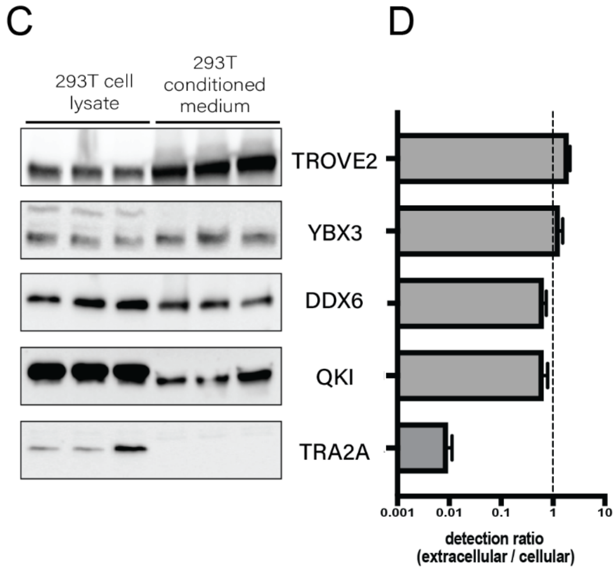

Figure S3

RBP detection in DiFi and HEK293T cell media, Related to Figure 3. A) Western blots performed on DiFi cell lysate, DiFi cell media, and HEK293T lysate with a media negative control. **B)** Summary table of the RBPs tested in the three cell types and mass spectrometry with corresponding detection success. **C)** Western blots performed in triplicate on HEK293T cell lysate and conditioned media for RBPs candidates computationally predicted in cell conditioned media. **D)** Ratio of detected signal between HEK293T conditioned medium and corresponding cell lysate blots. A value greater than one indicates a stronger detection of the RBP in the extracellular space, while below one shows a higher quantification intracellularly. Data are represented as mean + SD.

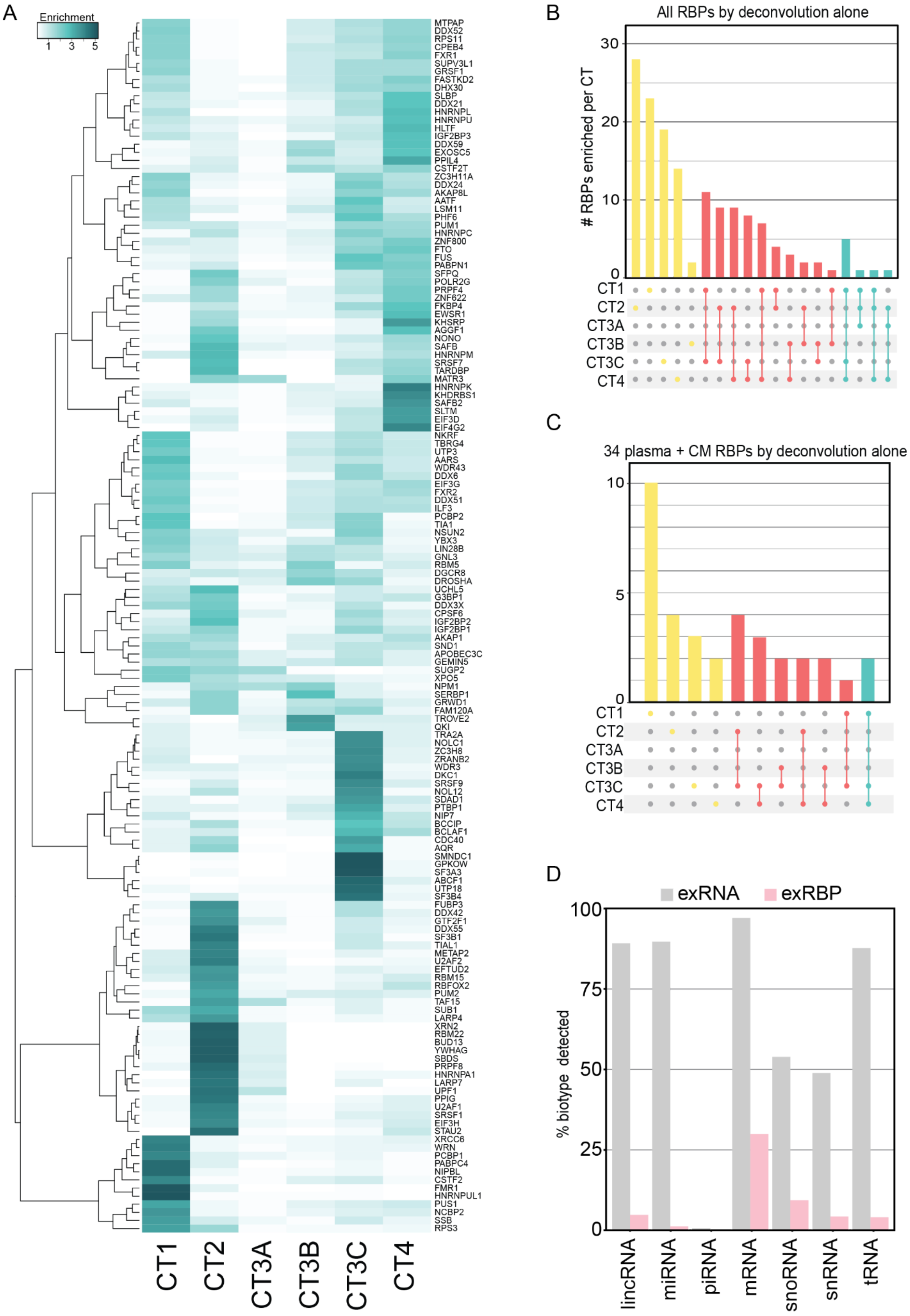
A
Enrichment
1 3 5
MTPAP
DDX52
RPS11
CPEB4
FXR1
SUPV3L1
GRSF1
FASTKD2
DHX30
SLBP
DDX21
HNRNPL
HNRNPU
HLTF
IGF2BP3
DDX59
EXOSC5
PPIL4
CSTF2T
ZC3H11A
DDX24
AKAP8L
AATF
LSM11
PHF6
PUM1
HNRNPC
ZNF800
FTO
FUS
PABPN1
SFPQ
POLR2G
PRPF4
ZNF622
FKBP4
EWSR1
KHSRP
AGGF1
NONO
SAFB
HNRNPM
SRSF7
TARDBP
MATR3
HNRNPK
KHDRBS1
SAFB2
SLTM
EIF3D
EIF4G2
NKRF
TBRG4
UTP3
AARS
WDR43
DDX6
EIF3G
FXR2
DDX51
ILF3
PCBP2
TIA1
NSUN2
YBX3
LIN28B
GNL3
RBM5
DGCR8
DROSHA
UCHL5
G3BP1
DDX3X
CPSF6
IGF2BP2
IGF2BP1
AKAP1
SND1
APOBEC3C
GEMIN5
SUGP2
XPO5
NPM1
SERBP1
GRWD1
FAM120A
TROVE2
QKI
TRA2A
NOLC1
ZC3H8
ZRANB2
WDR3
DKC1
SRSF9
NOL12
SDAD1
PTBP1
NIP7
BCCIP
BCLAF1
CDC40
AQR
SMNDC1
GPKOW
SF3A3
ABCF1
UTP18
SF3B4
FUBP3
DDX42
GTF2F1
DDX55
SF3B1
TIAL1
METAP2
U2AF2
EFTUD2
RBM15
RBFOX2
PUM2
TAF15
SUB1
LARP4
XRN2
RBM22
BUD13
YWHAG
SBDS
PRPF8
HNRNPA1
LARP7
UPF1
PPIG
U2AF1
SRSF1
EIF3H
STAU2
XRCC6
WRN
PCBP1
PABPC4
NIPBL
CSTF2
FMR1
HNRNPUL1
PUS1
NCBP2
SSB
RPS3
CT1 CT2 CT3A CT3B CT3C CT4
B
All RBPs by deconvolution alone
RBPs enriched per CT
0 10 20 30
CT1
CT2
CT3A
CT3B
CT3C
CT4
C
34 plasma + CM RBPs by deconvolution alone
0 5 10
CT1
CT2
CT3A
CT3B
CT3C
CT4
D
exRNA exRBP
% biotype detected
0 25 50 75 100
lincRNA
miRNA
piRNA
mRNA
snoRNA
snRNA
tRNA

Figure S4

XDec deconvolution of human biofluids allows assignment of RBP cargo to exRNA carrier types, Related to Figure 4. A) Heatmap of RBP CT enrichments with rows labeled. **B)**. Upset plot depicts how many RBPs are enriched for 1 or 2 different cargo types in deconvoluted profiles (regardless of enrichment in reference fractionated plasma/serum). **C)** Subset of Figure S4B for the 34 RBPs detected in bulk plasma and cell media (Figure S2C). **D)** Percent of biotype represented in exRNA Atlas with a coverage of 5 reads in at least one sample, vs those touched by at least 1 RBP site with a coverage of 5 reads in at least one sample in the studies used for deconvolution.

Table S1 – CT enrichments of all 150 RBPs, Related to Figure 4

	CT1	CT2	CT3A	CT3B	CT3C	CT4
AARS	1	0	0	0	0	0
AATF	0	0	0	0	1	0
ABCF1	0	0	0	0	1	0
AGGF1	0	1	0	0	0	1
AKAP1	1	1	0	0	0	0
AKAP8L	1	0	0	0	1	1
APOBEC3C	1	1	0	0	0	0
AQR	0	1	0	0	1	0
BCCIP	0	1	0	0	1	0
BCLAF1	0	0	0	0	1	1
BUD13	0	1	0	0	0	0
CDC40	0	1	0	0	1	0
CPEB4	1	0	0	0	0	1
CPSF6	0	1	0	0	1	0
CSTF2T	0	0	0	1	0	1
CSTF2	1	0	0	0	0	0
DDX21	0	0	0	0	0	1
DDX24	1	0	0	0	1	0
DDX3X	1	0	0	0	0	0
DDX42	0	1	0	0	0	0
DDX51	1	0	0	0	0	0
DDX52	1	0	0	0	0	1
DDX55	0	1	0	0	0	0
DDX59	0	0	0	1	0	1
DDX6	1	0	0	0	1	0
DGCR8	0	0	0	1	1	0
DHX30	1	0	0	0	1	1
DKC1	0	0	0	0	1	0
DROSHA	0	0	0	1	1	0
EFTUD2	1	0	0	0	0	0
EIF3D	0	0	0	0	0	1
EIF3G	1	0	0	0	0	1
EIF3H	0	1	0	0	0	0
EIF4G2	0	0	0	0	0	1
EWSR1	0	1	0	0	0	1
EXOSC5	0	0	0	1	0	1
FAM120A	0	1	0	0	1	0
FASTKD2	1	0	0	0	1	0
FKBP4	0	0	0	0	1	1
FMR1	1	0	0	0	0	0
FTO	0	0	0	0	1	1
FUBP3	0	1	0	0	0	0
FUS	0	0	0	0	1	1
FXR1	1	0	0	0	0	1
FXR2	1	0	0	0	0	1
G3BP1	0	1	0	0	0	0

GEMIN5	0	1	0	0	0	0
GNL3	1	0	0	0	0	0
GPKOW	0	0	0	0	1	0
GRSF1	1	0	0	0	1	1
GRWD1	0	1	0	0	1	0
GTF2F1	0	1	0	0	0	0
HLTF	0	0	0	0	0	1
HNRNPA1	0	1	0	0	0	0
HNRNPC	0	0	0	0	1	0
HNRNPK	0	0	0	0	0	1
HNRNPL	1	0	0	0	1	1
HNRNPM	1	1	0	0	0	1
HNRNPUL1	1	0	0	0	0	0
HNRNPU	0	0	0	0	0	1
IGF2BP1	0	1	0	0	1	0
IGF2BP2	0	1	0	0	0	0
IGF2BP3	0	0	0	0	0	1
ILF3	1	0	0	0	0	0
KHDRBS1	0	0	0	0	0	1
KHSRP	0	0	0	0	0	1
LARP4	0	1	0	0	0	0
LARP7	0	1	0	0	1	0
LIN28B	1	0	0	0	0	0
LSM11	1	0	0	0	1	0
MATR3	0	1	1	0	0	1
METAP2	0	1	0	0	0	0
MTPAP	1	0	0	0	0	1
NCBP2	1	0	0	0	0	0
NIP7	0	0	0	0	1	0
NIPBL	1	0	0	0	0	0
NKRF	1	0	0	0	0	0
NOL12	0	0	0	0	1	0
NOLC1	0	0	0	0	1	0
NONO	0	1	0	0	0	1
NPM1	0	1	0	1	0	0
NSUN2	1	0	0	0	1	0
PABPC4	1	0	0	0	0	0
PABPN1	0	0	0	0	1	1
PCBP1	1	0	0	0	0	0
PCBP2	1	0	0	0	1	0
PHF6	0	0	0	0	1	1
POLR2G	0	1	0	0	0	1
PPIG	0	1	0	0	0	0
PPIL4	0	0	0	0	0	1
PRPF4	0	1	0	0	0	1
PRPF8	0	1	0	0	0	0
PTBP1	0	0	0	0	1	0
PUM1	0	0	0	0	1	1
PUM2	0	1	0	0	0	0
PUS1	1	0	0	0	0	0
QKI	0	0	0	1	0	0
RBFOX2	0	1	0	0	0	0

RBM15	0	1	0	0	0	0
RBM22	0	1	0	0	0	0
RBM5	1	0	0	1	0	0
RPS11	1	0	0	0	0	1
RPS3	1	0	0	0	0	0
SAFB2	0	0	0	0	0	1
SAFB	0	1	0	0	0	1
SBDS	0	1	0	0	0	0
SDAD1	0	0	0	0	1	0
SERBP1	0	1	0	1	0	0
SF3A3	0	0	0	0	1	0
SF3B1	0	1	0	0	0	0
SF3B4	0	0	0	0	1	0
SFPQ	0	1	0	0	0	1
SLBP	0	0	0	0	0	1
SLTM	0	0	0	0	0	1
SMNDC1	0	0	0	0	1	0
SND1	1	0	0	0	0	0
SRSF1	0	1	0	0	0	0
SRSF7	0	0	0	0	0	1
SRSF9	0	0	0	0	1	0
SSB	1	0	0	0	0	0
STAU2	0	1	0	0	0	0
SUB1	1	1	0	0	0	0
SUGP2	1	1	1	0	0	0
SUPV3L1	1	0	0	0	1	1
TAF15	0	1	0	0	0	0
TARDBP	0	1	0	0	0	1
TBRG4	1	0	0	0	0	0
TIA1	1	0	0	0	1	0
TIAL1	0	1	0	0	0	0
TRA2A	0	0	0	0	1	0
TROVE2	0	0	0	1	0	0
U2AF1	0	1	0	0	0	0
U2AF2	0	1	0	0	0	0
UCHL5	1	1	0	0	0	0
UPF1	0	1	0	0	1	0
UTP18	0	0	0	0	1	0
UTP3	1	0	0	0	1	0
WDR3	0	0	0	0	1	0
WDR43	1	0	0	0	1	0
WRN	1	0	0	0	0	0
XPO5	1	0	0	0	0	0
XRCC6	1	0	0	0	0	0
XRN2	0	1	0	0	0	0
YBX3	1	0	0	0	1	0
YWHAG	0	1	0	0	0	0
ZC3H11A	1	0	0	0	1	0
ZC3H8	0	0	0	0	1	0
ZNF622	0	1	0	0	0	1
ZNF800	0	0	0	0	1	1
ZRANB2	0	0	0	0	1	0

Table S2 – CT enrichments of 47 consistently enriched RBPs, Related to Figure 4

	CT1	CT2	CT3A	CT3B	CT3C	CT4
APOBEC3C	1	0	0	0	0	0
CSTF2	1	0	0	0	0	0
FASTKD2	1	0	0	0	0	0
FMR1	1	0	0	0	0	0
GNL3	1	0	0	0	0	0
HNRNPM	1	0	0	0	0	0
LIN28B	1	0	0	0	0	0
SSB	1	0	0	0	0	0
SUB1	1	0	0	0	0	0
TIA1	1	0	0	0	1	0
YBX3	1	0	0	0	0	0
AKAP1	0	1	0	0	0	0
GEMIN5	0	1	0	0	0	0
HNRNPA1	0	1	0	0	0	0
RBM22	0	1	0	0	0	0
SERBP1	0	1	0	0	0	0
TAF15	0	1	0	0	0	0
U2AF2	0	1	0	0	0	0
AGGF1	0	0	0	0	0	1
POLR2G	0	0	0	0	0	1
PPIL4	0	0	0	0	0	1
SFPQ	0	0	0	0	0	1
DDX59	0	0	0	1	0	0
EXOSC5	0	0	0	1	0	0
QKI	0	0	0	1	0	0
TROVE2	0	0	0	1	0	0
ABCF1	0	0	0	0	1	0
AQR	0	0	0	0	1	0
CDC40	0	0	0	0	1	0
CPSF6	0	0	0	0	1	0
DKC1	0	0	0	0	1	0
FAM120A	0	0	0	0	1	0
LARP7	0	0	0	0	1	0
NIP7	0	0	0	0	1	0
NOL12	0	0	0	0	1	0
NOLC1	0	0	0	0	1	0
PABPN1	0	0	0	0	1	0
PTBP1	0	0	0	0	1	0
PUM1	0	0	0	0	1	0
SMNDC1	0	0	0	0	1	0
TRA2A	0	0	0	0	1	0
UPF1	0	0	0	0	1	0
UTP18	0	0	0	0	1	0
WDR3	0	0	0	0	1	0
WDR43	0	0	0	0	1	0
ZC3H8	0	0	0	0	1	0
ZRANB2	0	0	0	0	1	0

Table S3 – exRNA-atlas.org accessions of studies used in analysis, related to STAR Methods

Accession	Biofluids	Accession	Biofluids
EXR-DERLE1Dj6liR-AN	Plasma	EXR-DGALA1ECRXVs-AN	CSF
EXR-DGALA1V5h5va-AN	Plasma	EXR-DGALA1mUDpAe-AN	CSF
EXR-KVICK1oIp40e-AN	Plasma	EXR-DGALA1QDi9GG-AN	CSF
EXR-JJONE1wAX8tF-AN	Plasma	EXR-KJENS1sPlvS2-AN	CSF, Serum
EXR-KJENS17CZMbP-AN	Plasma	EXR-ANACC1S6lJ1C-AN	Plasma, Stool, Urine
EXR-SADAS1EXER1-AN	Plasma	EXR-JJONE1HC7DHb-AN	Urine
EXR-MTEWA1ZR3Xg6-AN	Plasma, Serum	EXR-JJONE1tHRzcU-AN	Urine
EXR-KJENS10lPClY-AN	Plasma	EXR-DWONG1qf3tcS-AN	Saliva
EXR-MTEWA1cHYLo6-AN	Plasma, Serum	EXR-IGHIR1HnDH6K-AN	Plasma, Serum
EXR-MTEWA1vczugX-AN	Plasma	EXR-JJONE1AcUuui-AN	Serum
EXR-KJENS1RID1-AN	Plasma, Urine, Saliva	EXR-LLAUR1s0A1mX-AN	Serum
EXR-AWEAV1lyeWbT-AN	Condition Medium	EXR-LLAUR1SFszrF-AN	Plasma, Serum
EXR-AKRIC157ITEl-AN	Condition Medium	EXR-TTUSC1gCrGDH-AN	Plasma, Serum
EXR-JFRAN1cDpMBU-AN	Condition Medium	EXR-KJENS12WGutU-AN	CSF
EXR-JFRAN16VDql8-AN	Condition Medium	EXR-KJENS1WBaSro-AN	CSF, Plasma
EXR-DGALA17UKOTF-AN	CSF	EXR-TPATE1OqELFf-AN	Plasma
EXR-DGALA1adMfMp-AN	CSF	EXR-MBITZ12SHVIr-AN	Urine
EXR-DGALA1B2gzMc-AN	CSF	EXR-SADAS1UJ0CzW-AN	Plasma
EXR-DERLE1PHASE1PROT-AN	Saliva, Sputum, Ovarian Follicle Fluid, Seminal Fluid, Amniotic Fluid, Plasma, Serum, CSF, BALF, Urine		

Table S4 – Detection of off strand reads, related to STAR Methods

	Av Wrong Strand		Av Wrong Strand		Av Wrong Strand
DDX42	0%	WDR43	0%	GTF2F1	1%
CDC40	0%	PUM2	0%	TBRG4	1%
NSUN2	0%	SLBP	0%	SERBP1	1%
GPKOW	0%	SF3B1	0%	HNRNPC	1%
SF3A3	0%	DROSHA	0%	EXOSC5	1%
GEMIN5	0%	RPS3	0%	PTBP1	1%
SMNDC1	0%	LIN28B	0%	AGGF1	1%
SSB	0%	ZNF622	0%	EIF4G2	1%
TAF15	0%	SND1	0%	SAFB2	1%

SF3B4	0%	DDX52	0%	DHX30	1%
TROVE2	0%	SUB1	0%	POLR2G	1%
AQR	0%	UTP3	0%	SAFB	1%
XRCC6	0%	EIF3G	0%	TIA1	1%
FKBP4	0%	QKI	0%	GNL3	1%
NPM1	0%	NIPBL	0%	ZC3H11A	2%
WRN	0%	NCBP2	0%	ABCF1	2%
DKC1	0%	UTP18	0%	FXR1	2%
HNRNPUL1	0%	NIP7	0%	FUS	2%
DDX21	0%	NKRF	0%	PRPF4	2%
NOLC1	0%	FAM120A	0%	PABPC4	2%
BCCIP	0%	CPSF6	0%	RBFOX2	2%
PUM1	0%	PPIG	0%	IGF2BP3	2%
EIF3D	0%	BUD13	0%	RPS11	2%
SUPV3L1	0%	PHF6	0%	HLTF	3%
WDR3	0%	XRN2	0%	RBM5	3%
EFTUD2	0%	GRWD1	0%	FMR1	3%
XPO5	0%	SRSF1	0%	LARP4	3%
DDX51	0%	ZNF800	0%	PABPN1	3%
RBM22	0%	TRA2A	0%	FUBP3	3%
APOBEC3C	0%	DDX24	0%	KHSRP	3%
ZRANB2	0%	BCLAF1	0%	HNRNPU	5%
DDX6	0%	SRSF9	0%	SUGP2	6%
NOL12	0%	YWHAG	0%	SLTM	9%
SRSF7	0%	IGF2BP1	0%	AKAP1	9%
LARP7	0%	FXR2	0%	SFPQ	14%
SBDS	0%	AARS	0%	TIAL1	15%
IGF2BP2	0%	HNRNPM	0%	EWSR1	16%
AKAP8L	0%	STAU2	0%	CPEB4	16%
METAP2	0%	FASTKD2	0%	ILF3	23%
FTO	0%	PCBP1	0%	HNRNPA1	23%
EIF3H	0%	TARDBP	0%	CSTF2	24%
PRPF8	0%	YBX3	0%	KHDRBS1	37%
ZC3H8	0%	UCHL5	0%	U2AF2	40%
DGCR8	0%	UPF1	0%	NONO	45%
SDAD1	0%	PUS1	0%	CSTF2T	45%
U2AF1	0%	GRSF1	0%	DDX59	48%
DDX55	0%	PPIL4	0%	HNRNPK	54%
MTPAP	0%	DDX3X	0%	HNRNPL	61%
AATF	0%	LSM11	0%	PCBP2	63%
G3BP1	0%	RBM15	1%	MATR3	88%
